# Prevalence and factors associated with dysmenorrhea in women at child bearing age in the Dschang Health District, West-Cameroon

**DOI:** 10.11604/pamj.2020.37.178.19693

**Published:** 2020-10-23

**Authors:** Axel Mbvoumi Nloh, Esther Ngadjui, Noël Vogue, Aimé Césaire Tetsatsi Momo, Georges Roméo Bonsou Fozin, Yannick Meli Yemeli, Pierre Watcho

**Affiliations:** 1 Faculty of Medicine and Pharmaceutical Sciences of the University of Dschang, Dschang, Cameroon,; 2 Research Unit of Animal Physiology and Phytopharmacology, Department of Animal Biology, Faculty of Science, University of Dschang, Dschang, Cameroon,; 3 Regional Delegation of Public Health of the Centre Region, Yaoundé, Cameroon

**Keywords:** Prevalence, dysmenorrhea, associated factors, Dschang Health District

## Abstract

**Introduction:**

dysmenorrhea is a painful phenomenon at the pelvis region preceding or following menstruation. Dysmenorrhea accounts among the most frequent problem of women at child bearing age and affects 45% to 95% of them. According to the WHO, 16.8 to 81% of women are affected by dysmenorrhea. The present study was carried out at the Dschang Health District in order to determine the prevalence of dysmenorrhea and associated factors among women at child bearing age.

**Methods:**

a transversal community-based study was carried out from March to June 2018. Information regarding socio-demographic features, prevalence, factors associated with the dysmenorrhea and the effect of dysmenorrhea on daily activities were collected using structured questionnaire and data were analyzed using Epi Info version 7.1.3.3 Software.

**Results:**

a total of 637 women aged 12 to 50 years were interviewed in the present study. The mean body mass index was 25.94 with an average weight of 66.41 kilogram. Fifty six point twenty percent (56.20%) of participants had dysmenorrhea. From all risks factors fund only the normal body mass index (OR = 3.08, P-value = 0.01) having a significant association with the occurrence of dysmenorrhea. Daily activities were affected in 73.25% of participants dysmenorrheic and those who had some episodes of dysmenorrhea.

**Conclusion:**

the present study showed that more than a half of respondents were dysmenorrheic and several factors were associated with this pathology. This study also suggests that dysmenorrhea have a negative impact on the daily activities of women at child bearing age.

## Introduction

Dysmenorrhea can be defined as severe, painful and cramp-like sensation at the lower abdomen during menstruation [1]. It may be categorized into primary and secondary dysmenorrhea. Primary dysmenorrhea is menstrual pain without pelvic pathology, with onset typically just after menarche. Secondary dysmenorrhea is menstrual pain associated with a causative pathology [2]. To explain the etiology of primary dysmenorrhea the most accepted theory is the over production of prostaglandins in endometrium during ovulatory cycles [3]. Prostaglandin stimulate the myometrial contractions and local vasoconstrictions that cause the menstrual effluent to be expelled from the uterine cavity [4]. However, secondary dysmenorrhea can be caused by disorders such as endometriosis, pelvic inflammatory disease, intrauterine adhesions or cervical stenosis [5]. There is a wide variation in the estimate of dysmenorrhea from studies around the world.

According to Proctor and Farquhar, the prevalence of dysmenorrhea varies from 45% to 95% depending on the age group [6]. Moreover, the WHO found a prevalence of 16.8 to 81% of women suffering from dysmenorrhea [7]. Dysmenorrhea associated to pelvic pain is one of the most common gynecologic complaints in Women of Child Bearing Age (WCBA) [8, 9]. It is commonly associated to a high rate of absenteeism and low productivity in daily activities. To this important socio-economic dimension is added the psychological impact of the repetitive pain [10]. Dysmenorrhea is therefore a public health problem [4, 11]. In Cameroon, dysmenorrhea is not neglected but very few studies have been done on, especially those relating to the prevalence and associated factors. In accordance with the Sustainable Development Goal (SDG) number 3 which aimed at empowering people to live healthy lives and promoting the well-being of all and at all ages [12], the present study was undertaken to determine the prevalence and factors associated with dysmenorrhea in women of childbearing age in the Dschang Health District (DHD).

## Methods

**Study design:** a transversal (descriptive and analytic), community-based study was conducted from March to June 2018 in the DHD. Data were collected using a four-part structured questionnaire. An initial survey tested the questionnaire and adapted it to the study population. In this study, the target population was women at child bearing age in DHD and the source population women at child bearing age from 8 Health Areas (HA) of DHD.

### Selection Criteria

**Inclusion criteria:** included in this study were all girls who experiment menarche and non-menopausal women, aged from 12 to 50 years and agreed to participate.

**Exclusion criteria:** were excluded from this study, all those expressing the need to stop the interview during data collection.

### Sampling

**Sample size:** sample size was calculated by assuming 95% confidence level, 5% absolute precision or marginal error. Having no prevalence of dysmenorrhea in women of reproductive age in Cameroon in general, and in the DHD in particular, the prevalence of 75% from the study conducted by El-Gilany in Egypt was used [13]. The minimum sample size was then estimated according to Lwanga and Lemeshow [14] as follows:

$$n=\frac{p\left(1-\text{p}\right)Z^{2}}{d^{2}}$$

n = minimum sample size, Z = 95% confidence level (typical value of 1.96), P = prevalence of dysmenorrhea, d = accuracy of the study with margin of error at 5% (typical value 0.05). The minimum value of our sample size was: n = 1.96 x 1.96 x 0.75 x 0.25 / 0.05 x 0.05 = 288.12 ≈ 288 WCBA, thus a cluster effect of 2 gave a sample size for this study of: n = 288 * 2 = 576 WCBA. To account for non-respondents, 10% (non-response rate) was added to this sample size: n + 10% = 576+ (576 x 10/100) = 633.6 ≈ 634 WCBA. The number of clusters being 30, the number of people per cluster was obtained by the following formula:

$$\text{Population by cluster}=\frac{Sampledpopulation}{numberofcluster}$$

Population by cluster = 634/30 = 21.13 ≈ 21 WCBA

**Sampling method:** the sampling method used in this study was the random or probabilistic method, precisely a two (2) degree sampling. The first degree was to choose the number of health areas in the DHD and the second was the selection of 30 villages/quarters to be investigated.

**Data collection and treatment:** a face to face household questionnaire was administered to participants. Questions regarding socio-demographic features, prevalence, factors associated with the dysmenorrhea and the effect of dysmenorrhea on daily activities of WCBA in DHD were included on this questionnaire. Patients´ weight and height were collected for the determination of Body Mass Index (BMI).

**Data analysis:** data were analyzed using Epi Info version 7.1.3.3 Software. The prevalence of dysmenorrhea was estimated and the simple logistic regressions of the variables was applied to identify risks factors. After obtaining raw OR and P from simple logistic regressions, the multiple logistic regression of risks factors was done to adjust their ORs and P. The test was significant at P ≤ 0.05.

**Ethics considerations:** ethical clearance N° 2018/05/1035 / CE / CNERSH / SP was obtained from the National Committee of Ethics of Research for Human Health of Cameroon. An informed consent form or an informed consent read and signed by respondents or parents of minor respondents. Research authorizations had also been obtained from the administrative and traditional authorities. Data confidentiality was firmly followed.

## Results

**Socio-demographic characteristics of participants:** during this study, 637 WCBA were interviewed, the age of the respondents ranged from 12 to 50 years. The average age of respondents was 24 years (SD 10.83), 284 (44.58%) WCBA were between 12 and 19 years of age. The minimum weight of the respondents was 38 kg while the maximum was 120 kg with an average of 66.49 kg (SD 12.76). The average height of respondents was 160.03 centimeters (SD 5.81). In the study frequency of respondents based on their BMI show that 298 (46.78%) of WCBA had a normal BMI and 6 (0.94%) had a BMI less than 24.9. This results are showing in Table 1. The distribution of respondents according to their marital status shows that 438 (68.76%) WCBA were single, 59 (24.96%) were married, 25 (3.92%) living in concubine, 6 (0.94%) were divorced/separate and 9 (1.41%) were widow. Concerning medical consultation due to their dysmenorrhea only 99 (25.71%) from 385 dysmenorrheic WCBA and the one who experienced dysmenorrhea before they stopped consulted a medical doctor or health staff.

**Table 1:** socio-demographic characters and some characteristics of the menstrual cycle of the respondents

Variables (n=637)	Mean	SD	All WCBA (%)	12-19 years (%)	20-25 years (%)	26-34 years (%)	35-50 years (%)
**Socio demographic characters**							
Age	24.54	10.75	**-**	284 (44.58)	154 (24.18)	65 (10.20)	134 (21.04)
Weight	66.49	12.76	**-**	**-**	**-**	**-**	**-**
Height	160.03	5.81	**-**	**-**	**-**	**-**	**-**
Normal BMI	**-**	**-**	298 (46.78)	175 (61.62)	75 (48.70)	18 (27.69)	30 (22.39)
Overweight	**-**	**-**	229 (35.95)	89 (31.34)	60 (38.96)	26 (40.00)	54 (40.30)
Obese	**-**	**-**	90 (14.13)	15 (5.28)	16 (10.39)	20 (30.77)	39 (29.10)
Severe obesity	**-**	**-**	14 (2.20)	2 (0.70)	**-**	1 (1.54)	11 (8.21)
Lean	**-**	**-**	6 (0.94)	3 (1.06)	3 (1.95)	**-**	**-**
Low financial income	**-**	**-**	336 (52.75)	**-**	**-**	**-**	**-**
Average income	**-**	**-**	293 (46.00)	**-**	**-**	**-**	**-**
High financial income	**-**	**-**	8 (1.26)	**-**	**-**	**-**	**-**
**Menstrual cycle characteristics**							
Age at menarche	13.78	1.58	**-**	**-**	**-**	**-**	**-**
Bleeding between 4 and 6 days	**-**	**-**	450 (70.64)	**-**	**-**	**-**	**-**
Bleeding less than 4 days	**-**	**-**	161 (2.27)	**-**	**-**	**-**	**-**
Bleeding less than 4 days	**-**	**-**	26 (4.08)	**-**	**-**	**-**	**-**
Moderate bleeding	**-**	**-**	422 (66.24)	**-**	**-**	**-**	**-**
Significant bleeding	**-**	**-**	134 (21.03)	**-**	**-**	**-**	**-**
low bleeding	**-**	**-**	81 (12.71)	**-**	**-**	**-**	**-**
Moderate pain	**-**	**-**	187 (48.57)	**-**	**-**	**-**	**-**
Severe pain	**-**	**-**	155 (40.26)	**-**	**-**	**-**	**-**
Mild pain	**-**	**-**	43 (11.17)	**-**	**-**	**-**	**-**

**Characteristics of the menstrual cycle of the respondents:** the study revealed that 52.43% of WCBA had their first menses between 13 and 14 years. The Table 1 shows that average age at menarche among the respondents was 13.78 years (SD 1.58). Concerning the duration of bleeding during menstruation 450 (70.64%) of respondents bleeding from 4 to 6 days.

**Figure 1:**
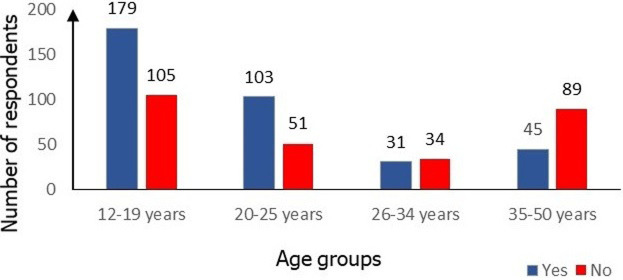
prevalence of dysmenorrhea according to age group

**Prevalence of dysmenorrhea in WCBA of DHD:** the prevalence of dysmenorrhea among WCBA who participated in this study is shown in Figure 1. It can be noted that 358 (56.20%) suffered from dysmenorrhea. The prevalence of dysmenorrhea by age group is shown in the figure. From this figure we note that 103 (66.88 %) dysmenorrheic respondents were aged 20-25 years old.

**Characteristics of dysmenorrhea among respondents:** the intensity of perceived pain during their dysmenorrhea is showing in Table 1. In this table 187 (48.57%) reported having moderate pain during menses. Concerning the distribution of WCBA according the duration of pain, from 385 dysmenorrheic WCBA and the one who experienced dysmenorrhea before they stopped, 100 (25.97%) experienced dysmenorrhea during one day and 179 (46.49%) during two days. During the dysmenorrhea some factors could increase the intensity of pain, the study show that of the 385 dysmenorrheal WCBA and the one who experienced dysmenorrhea before they stopped, 104 (27.01%) had an increase of pain when they were stressed, 64 (16.62%) during a school examination period, 50 (12.98%) after school failure, 39 (10.12%) in a family dispute and 27 (7.01%) had an increase in the intensity of pain when they went through a love disappointment.

**Signs accompanying dysmenorrhea:** from 358 WCBA dysmenorrheal 180 (50.28%) of women felt tired during dysmenorrhea, 174 (48.60%) had abdominal bloating, 164 (45.81%) were irritable, 149 (41.62%) had headache, 137 (38.27%) nausea, 115 (32.12%) dizziness, 110 (30.73%) reported insomnia, 87 (23.30%) reported breast pain, 63 (17.60%) experienced fever, 37 (10.34%) reported diarrhea, 33 (9.22%) had vomit and 46 12,85% (12.85%) had no signs accompanying dysmenorrhea.

**Factors associated with dysmenorrhea:** the simple logistic regressions of the variables are presented in Table 2. This table shows that risks factors which the highest OR were having a dysmenorrheic mother (OR = 13.56), having a sexual intercourse before the onset of dysmenorrhea (OR = 1.78) and having the BMI between 18.5 and 24.9 (OR = 1.71). After adjustment of risks factors only having BMI between 18.5 and 24.9 have a significant association with the occurrence of dysmenorrhea in WCBA. The multiple logistic regression of risks factors are shown in Table 3.

**Table 2:** simple logistic regressions of factors associated with dysmenorrhea

Factors	OR	CI	P-value
Having a dysmenorrheic mother	13.56	4.17-44.09	0.00*
Living with a smoker	1.13	0.71-1.79	0.60
Menarche between 13 and 14 years	1.08	0.79-1.49	0.59
Menarche ≤12 years	1.50	1.00-2.24	0.04*
Menarche >14	0.64	0.45-0.91	0.01*
Duration of menstrual period between 4 and 6 days	1.55	1.09-2.20	0.01*
Duration of menstrual period <4 days	0.57	0.39-0.82	0.00*
Duration of menstrual period >6 days	1.00	0.48-2.05	0.99
Significant bleeding	1.63	1.63-2.42	0.01*
Low bleeding	0.92	0.57-1.49	0.75
Moderate bleeding	0.74	0.53-1.04	0.08
Having lower abdomen pain without menses	0.54	0.23-1.29	0.16
Having urinary pain	1.44	0.33-6.36	0.62
BMI between 18.5 and 24.9	1.71	1.25-2.35	0.00*
BMI between 24.9 and 29.9	0.98	0.70-1.35	0.90
BMI <24.9	1.56	0.28-8.60	0.60
BMI between 29.9 and 40	0.43	0.27-0.68	0.00*
BMI ≥ 40	0.48	0.18-1.27	0.14
To have a child	0.35	0.25-0.49	0.00*
Having a sexual intercourse before the onset of dysmenorrhea	1.78	0.40-7.79	0.44
Consult a Heath worker	0.42	0.19-0.94	0.03*

P-value* = significant value less than 0.05

**Table 3:** multiple logistic regression of risks factors

Risks factors	OR	CI	P-Value
Having a dysmenorrheic mother	3.67	0.47-29.74	0.21
Living with a smoker	1.47	0.40-5.36	0.55
Duration of menstrual period between 4 and 6 days	1.90	0.77-4.64	0.15
Having urinary pain	1.15	0.24-5.48	0.85
Having a sexual intercourse before the onset of dysmenorrhea	2.73	0.56-13.19	0.21
Menarche between 13 and 14 years	2.33	0.95-5.70	0.06
Menarche less or equal than 12 years	2.57	0.76-8.68	0.12
BMI between 18.5 and 24.9	3.08	1.27-7.43	0.01*
Significant bleeding	0.66	0.26-1.65	0.38

P-value*= significant value less than 0.05

**Effect of dysmenorrhea on the daily activities of respondents:** the effect of dysmenorrhea in the daily activities of respondents is shows in Table 4. Out of the 385 dysmenorrheal WCBA and the one who experienced dysmenorrhea before they stopped 282 (73.25%) declared disturbed daily activities during their pain menses while 103 (26.75%) who said their daily activities were not affected.

**Table 4:** prevalence of dysmenorrhea and distribution of WCBA according to whether their activities are disturbed by dysmenorrhea

	Yes	No
**Dysmenorrhea**	56.20%	43.80%
**Activities disturbed**	73.25%	26.75%

## Discussion

The present community-based study was conducted from March to June 2018 in the DHD and aimed at determining the prevalence and factors associated with dysmenorrhea in WCBA. A total of 637 WCBA were interviewed and results showed that 56.20% of the respondents were dysmenorrheic. This prevalence is lower than 58.1% found in 2004 in New Zealand [15] and higher than 45% found in 2008 among Nigerian college women [16]. The variation in the prevalence of dysmenorrhea can be attributed to the lack of a universally accepted definition of dysmenorrhea [17] and the variation of socio-cultural and geographical data [18]. Dysmenorrhea affected all group of WCBA from respondent, 63.02% adolescent were dysmenorrheic, prevalence lower than of 65.8% found by Houston [19] and higher than 62.8% found by Rodrigues [20]. Numerous factors may be associated with the occurrence of dysmenorrhea, the one with the highest OR was having a dysmenorrheic mother OR = 3.67 and P = 0.21 are similar to the OR = 2.49 and a P-value = 0.05 obtained by Jaiprakash [21]. Dysmenorrhea can negatively affect women education and socio-professional activities. In this study, 73.25% of the dysmenorrheic had their daily activities disturbed. This frequency is close to the frequency of 86.31% obtained by Narring in Swaziland [9]. Despite these results, the study presents some limits such as; absence of blood analyses of dysmenorrheic respondents in order to evaluate the blood levels of prostaglandin, leukotriene, arginine and vasopressin.

## Conclusion

At the end of this study, more than half of the respondents suffered from dysmenorrhea. Factors associated with the onset of dysmenorrhea included but only have BMI between 18.5 and 24.9 have a significant association. Dysmenorrhea has a significant impact on WCBA daily activities, 73.25% of WCBA dysmenorrheal and the one who experienced dysmenorrhea before they stopped in the DHD saw their school, work, social and economic activities disrupted.

### What is known about this topic

- Dysmenorrhea are categorized in two types, primary and secondary dysmenorrhea;
- Etiology of primary dysmenorrhea is the over production of prostaglandins in endometrium and the secondary dysmenorrhea can be caused by disorders such as endometriosis and pelvic inflammatory disease;
- WHO found a prevalence of 16.8 to 81% of women suffering from dysmenorrhea.

### What this study adds

- To determine the prevalence of dysmenorrhea in WCBA of the DHD;
- To identify the factors associated with the occurrence of dysmenorrhea;
- Finally to determine the frequency of WCBA having their activities disturbed by dysmenorrhea.
